# Clinical trials of CAR-T cells in China

**DOI:** 10.1186/s13045-017-0535-7

**Published:** 2017-10-23

**Authors:** Bingshan Liu, Yongping Song, Delong Liu

**Affiliations:** 1 School of Basic Medical Sciences and The Affiliated Cancer Hospital of Zhengzhou University, Zhengzhou, China; 2 Henan Cancer Hospital and The Affiliated Cancer Hospital of Zhengzhou University, 127 Dongming Road, Zhengzhou, 450008 China

## Abstract

Novel immunotherapeutic agents targeting tumor-site microenvironment are revolutionizing cancer therapy. Chimeric antigen receptor (CAR)-engineered T cells are widely studied for cancer immunotherapy. CD19-specific CAR-T cells, tisagenlecleucel, have been recently approved for clinical application. Ongoing clinical trials are testing CAR designs directed at novel targets involved in hematological and solid malignancies. In addition to trials of single-target CAR-T cells, simultaneous and sequential CAR-T cells are being studied for clinical applications. Multi-target CAR-engineered T cells are also entering clinical trials. T cell receptor-engineered CAR-T and universal CAR-T cells represent new frontiers in CAR-T cell development. In this study, we analyzed the characteristics of CAR constructs and registered clinical trials of CAR-T cells in China and provided a quick glimpse of the landscape of CAR-T studies in China.

## Background

Novel immunotherapeutic agents targeting CTLA-4, programmed cell death-1 protein receptor (PD-1), and the ligand PD-L1 are revolutionizing cancer therapy [1–7]. Cancer immunotherapy by re-igniting T cells through blocking PD-1 and PD-L1 is highly potent in a variety of malignancies [8–12]. Allogeneic hematopoietic stem cell transplantation has been proven to be a curative immunotherapy for leukemia though with significant toxicities [13–18]. Autologous T cells with re-engineered chimeric antigen receptors (CAR-T) have been successfully used for leukemia and lymphoma without graft-vs-host diseases [19–25]. The first such product, tisagenlecleucel, has recently been approved for clinical therapy of refractory B cell acute lymphoblastic lymphoma (ALL). More and more clinical trials of CAR-T cells are being done throughout the world [26–38].

In recent years, more and more clinical trials from China are being done and registered in ClinicalTrials.gov. CAR-T cells have become a major source of cellular immunotherapy in China. This study summarized the CAR-T clinical trials being conducted in China and provided a quick glimpse of the landscape of CAR-T studies in China.

## Methods

We searched ClinicalTrials.gov using keywords “CAR T,” “CAR-T,” “chimeric antigen receptor,” “adoptive therapy,” “third generation chimeric,” and “fourth generation chimeric”; country: China. All relevant trials registered at the ClinicalTrials.gov prior to July 18, 2017, were included in the analysis. One trial was excluded (NCT03121625) because the target antigen was not disclosed. A search of the PubMed database was also done to include those trials and cases that have been published.

## Results

### Distribution of CAR-T trials in China

Currently, there are 121 trials reported and/or registered at ClinicalTrials.gov from China (Table 1). The trials are mainly carried out in leading hospitals from Beijing, Shanghai, Guangzhou, and Chongqing. CAR-T trials are started in hospitals throughout China. In this study, to avoid duplication of trials that can lead to miscalculation, those trials in Chinese registries were not included. It is possible that the number of institutions carrying out CAR-T trials will increase at a slower pace once regulatory policies are in place. We believe these CAR-T cells should be regulated as drugs [39].

**Table 1:** Distribution of clinical trials with CAR-T cells in China

Beijing	30
Shanghai	22
Guangdong	20
Chongqing	15
Jiangsu	13
Others	21

### Chimeric antigen receptors, vectors, and co-stimulatory molecules used in the CAR constructs

T cell receptors (TCRs) are engineered by incorporating a specific antigen-targeting element and CD3 element to form a completely novel TCR structure, the chimeric antigen receptor (CAR) [35, 40]. In addition, several co-stimulating sequences have been used to facilitate the expansion of the CAR-T cells [41]. CAR-engineered T lymphocytes have been in active clinical development to treat patients with advanced leukemia, lymphoma, and solid tumors [42–45].

One of the major hurdles in CAR-targeted cellular therapy has been the limited cell dose due to the lack of adequate in vivo cell expansion. Co-stimulatory signals can enhance immune responses of effector T cells [46]. Inducible co-stimulatory signal (ICOS), 4-1BB (CD137), CD28, OX40 (CD134), CD27, and DAP10, along with CD3ζ, have been investigated [31, 47–50]. Among these, 4-1BB (CD137), CD28, and CD3ζ are the most commonly used COS elements in the CARs (Tables 2, 3, and 4) [51, 52].

**Table 2:** Clinical trials of CD19-directed CAR-T cells in China

Target antigen	Diseases	CAR	Vector	NCT no.
CD19	Leukemia, lymphoma	4-1BB- CD3ζ	RV	NCT01864889
CD19	B cell malignancies	CD28, CD137, CD27	LV	NCT03050190
CD19	MCL	4-1BB-CD3ζ	RV	NCT02081937
CD19	Leukemia	NA	NA	NCT03142646
CD19	B cell lymphomas	CD27-CD3ζ	LV	NCT02247609
CD19	Leukemia, lymphoma	NA	NA	NCT02349698
CD19	Elderly relapsed/refractory B cell ALL	NA	NA	NCT02799550
CD19	Leukemia, lymphoma	NA	NA	NCT02537977
CD19	B cell leukemia	NA	NA	NCT02644655
CD19	B cell leukemia and lymphoma	NA	NA	NCT02813837
CD19	B cell lymphoma	NA	NA	NCT02547948
CD19	B cell lymphoma	CD28-CD3ζ	RV	NCT02652910
CD19	Leukemia, lymphoma	CD28, CD3ζ	LV or RV	NCT02456350
CD19	Recurrent or refractory acute non-T-lymphocyte leukemia	NA	NA	NCT02735291
CD19	Lymphoma	NA	NA	NCT02728882
CD19	Leukemia, lymphoma	NA	NA	NCT02546739
CD19	B cell lymphomas	NA	NA	NCT02842138
CD19	ALL	NA	NA	NCT02810223
CD19	ALL	CD28-CD137-CD3ζ	LV	NCT02186860
CD19	B cell leukemia, B cell lymphoma	CD3ζ, CD28, and 4-1BB	LV	NCT02963038
CD19	NHL	TCRζ, 4-1BB	LV	NCT03029338
CD19	B cell ALL	TCRζ, 4-1BB	LV	NCT02975687
CD19	B cell leukemia and lymphoma	NA	LV	NCT02933775
CD19	B cell leukemia	4-1BB	LV	NCT02672501
CD19	Central nervous system B cell acute lymphocytic leukemia	NA	NA	NCT03064269
CD19	ALL	4-1BB	LV	NCT02965092
CD19	Acute leukemia	NA	NA	NCT02822326
CD19	Leukemia, lymphoma	CD28 or 4-1BB and a CD3ζ	LV or RV	NCT03076437
CD19	Leukemia and lymphoma	NA	NA	NCT02851589
CD19	Leukemia and lymphoma	NA	NA	NCT02819583
CD19	DLBCL	NA	LV	NCT02976857
CD19	Recurrent or refractory B cell malignancy	NA	NA	NCT02782351
CD19	Leukemia and lymphoma	TCRz-CD28, TCRz-CD137	NA	NCT02685670
CD19	B cell lymphoma	4-1BB, CD3ζ	NA	NCT03101709
CD19	ALL	NA	NA	NCT02924753
CD19	ALL	NA	NA	NCT03027739
CD19	B cell leukemia	NA	LV	NCT02968472
CD19	B cell lymphoma	CD28ζ	NA	NCT02992834
CD19	AML	NA	NA	NCT03018093
CD19	Systemic lupus erythematosus	4-1BB	LV	NCT03030976
CD19	NHL	NA	LV	NCT03154775
CD19	Lymphoma	NA	NA	NCT03086954
CD19	ALL, CLL, lymphoma	CD28 or 4-1BB and CD3ζ	NA	NCT03191773
CD19	B cell lymphoma	4-1BB-CD28-CD3	NA	NCT03146533
CD19	Leukemia	NA	NA	NCT03173417
CD19	Relapsed or refractory B cell lymphoma	4-1BB	LV	NCT03208556
CD19	B cell leukemia and lymphoma			NCT03166878
CD19	B cell lymphoma	NA	NA	NCT03118180
CD19 or CD20	Relapse/refractory B cell malignancies	NA	LV	NCT02846584
CD19 and CD20	DLBCL	NA	NA	NCT02737085
CD19 and CD22	Hematopoietic/lymphoid cancer	TCRζ, 4-1BB	NA	NCT02903810
CD19/CD20	B cell leukemia and lymphoma	CD3ζ, 4-1BB-CD3ζ	RV	NCT03097770
CD19/CD22	B cell malignancy	NA	RV	NCT03185494
CD19/CD22	B cell leukemia, B cell lymphoma	NA	LV	NCT03098355
CD19/CD20/CD22/CD30	B-NHL	NA	NA	NCT03196830
CD19/CD20	B cell malignancy	NA	NA	NCT03207178
CD19 and CD20/CD22/CD38/CD123	B cell malignancy	NA	LV	NCT03125577

*AMMS* Academy Military Medical Sciences, *ALL* acute lymphoblastic leukemia, *AML* acute myeloid leukemia, *BCMA* B cell maturation antigen, *CTX* cyclophosphamide, *DLBCL* diffuse large B cell lymphoma, *FLU* fludarabine, *HL* Hodgkin’s lymphoma, *LV* lentiviral, *MCL* mantle cell lymphoma, *N*A not available, *NHL* non-Hodgkin lymphoma, *RV* retroviral, *TCM* traditional Chinese medicine

**Table 3:** Clinical trials of CAR-T cells targeting non-CD19 antigens in China

Target Antigen	Disease	CAR	Vector	NCT no.
CD20	Lymphoma	4-1BB-CD3ζ	LV	NCT01735604
CD20	B cell lymphoma	CD3ζ and CD28	RV	NCT02965157
CD20	B cell malignancies	NA	NA	NCT02710149
CD22	CD19-refractory or resistant lymphoma	TCRζ, 4-1BB	RV	NCT02721407
CD22	Recurrent or refractory B cell malignancy	NA	NA	NCT02794961
CD22	B cell malignancies	NA	NA	NCT02935153
CD30	Lymphoma	NA	LV	NCT02274584
CD30	HL, NHL	NA	NA	NCT02259556
CD30	Lymphocyte malignancies	NA	NA	NCT02958410
CD33	AML	4-1BB-CD3ζ	RV	NCT01864902
CD33	AML	NA	NA	NCT02799680
CD33	Myeloid malignancies	NA	NA	NCT02958397
BCMA	B cell malignancies	NA	NA	NCT02954445
BCMA	Multiple myeloma	TCRζ, 4-1-BB	RV	NCT03093168
CD123	Leukemia	NA	NA	NCT02937103
CD123	AML recurred after allo-HSCT	41BB-CD3ζ	NA	NCT03114670
CD138	Multiple myeloma	4-1BB-CD3ζ	RV	NCT01886976
CD138/BCMA	Multiple myeloma	NA	NA	NCT03196414
Lewis-Y	Myeloid malignancies	NA	NA	NCT02958384

*AMMS* Academy of Military Medical Sciences, *ALL* acute lymphoblastic leukemia, *AML* acute myeloid leukemia, *BCMA* B cell maturation antigen, *CTX* cyclophosphamide, *FLU* fludarabine, *HL* Hodgkin’s lymphoma, *LV* lentiviral, *MCL* mantle cell lymphoma, *NA* not available, *NHL* non-Hodgkin lymphoma, *RV* retroviral, *TCM* traditional Chinese medicine

**Table 4:** Clinical trials of CAR-T cells for solid tumors in China

Target antigens	Diseases	CAR	Vector	NCT no.
GPC3	Hepatocellular carcinoma	NA	NA	NCT02723942
GPC3	Hepatocellular carcinoma	CD3ζ, CD28, and 4-1BB	NA	NCT02395250
GPC3	Lung squamous cell carcinoma	NA	LV	NCT02876978
GPC3	Hepatocellular carcinoma and liver metastases	4-1BB	NA	NCT02715362
GPC3	Hepatocellular carcinoma	4-1BB	NA	NCT03130712
GPC3	Advanced hepatocellular carcinoma	4-1BB-CD3ζ	RV	NCT03084380
GPC3	Hepatocellular carcinoma, squamous cell lung cancer	NA	NA	NCT03198546
GPC3	Hepatocellular carcinoma	NA	LV	NCT03146234
GPC3, mesothelin, CEA	Hepatocellular, pancreatic cancer, colorectal cancer	NA	LV	NCT02959151
Mesothelin	Malignant mesothelioma, pancreatic Cancer, ovarian tumor, triple-negative breast cancer, endometrial cancer, other mesothelin-positive tumors	4-1BB-CD3ζ	RV	NCT02580747
Mesothelin	Recurrent or metastatic malignant tumors	NA	NA	NCT02930993
Mesothelin	Pancreatic cancer and pancreatic ductal a denocarcinoma	4-1BB	NA	NCT02706782
Mesothelin	Solid tumor, adult advanced cancer	NA	NA	NCT03030001
Mesothelin	Advanced solid tumor	NA	NA	NCT03182803
EpCAM	Liver neoplasms	NA	NA	NCT02729493
EpCAM	Stomach neoplasms	NA	NA	NCT02725125
EpCAM	Nasopharyngeal carcinoma and breast cancer	NA	LV	NCT02915445
EpCAM	Colon cancer, esophageal carcinoma, pancreatic cancer, prostate cancer, gastric cancer, hepatic carcinoma	CD3ζ, CD28	LV	NCT03013712
GD2	Neuroblastoma	NA	LV	NCT02765243
GD2	Relapsed or refractory neuroblastoma	NA	NA	NCT02919046
GD2	Solid tumor	NA	LV	NCT02992210
HER-2	Advanced HER-2-positive solid tumors	CD3ζ, 4-1BB-CD3ζ	NA	NCT01935843
HER-2	Breast cancer	CD28-CD3ζ	RV	NCT02547961
HER-2	Breast cancer, ovarian cancer, lung cancer, gastric cancer, glioma, pancreatic cancer	NA	NA	NCT02713984
EGFR	Advanced EGFR-positive solid tumors	4-1BB-CD3ζ	LV	NCT01869166
EGFR	Advanced solid tumor	NA	NA	NCT03182816
EGFR	Colorectal cancer	4-1BB-CD28-CD3	NA	NCT03152435
EGFRvIII	Recurrent glioblastoma multiform	NA	LV	NCT02844062
EGFRvIII	Glioblastoma multiform	NA	NA	NCT03170141
MUC1	Malignant glioma of brain, colorectal carcinoma, gastric carcinoma	NA	NA	NCT02617134
MUC1	Advanced refractory solid tumor (hepatocellular carcinoma, NSCLC, pancreatic carcinoma, triple-negative invasive breast carcinoma)	CD28-4-1BB- CD3ζ	LV	NCT02587689
MUC1	Advanced solid tumor	NA	NA	NCT03179007
CEA	Lung cancer, colorectal cancer, gastric cancer, breast cancer, pancreatic cancer	NA	NA	NCT02349724
EphA2	EphA2-positive malignant glioma	NA	NA	NCT02575261
LMP1	Nasopharyngeal neoplasms	NA	NA	NCT02980315
MG7	Liver metastases	4-1BB	NA	NCT02862704
CD133	Liver cancer, pancreatic cancer, brain tumor, breast cancer, ovarian tumor, colorectal cancer, ALL, AML	CD3ζ, 4-1BB-CD3ζ	RV	NCT02541370
HerinCAR-PD1	Advanced malignancies	NA	NA	NCT02873390
HerinCAR-PD1	Advanced solid tumor (lung, liver, and stomach)	NA	NA	NCT02862028
PD-L1 CSR	Glioblastoma multiform	NA	NA	NCT02937844
NY-ESO-1	Advanced NSCLC	NA	LV	NCT03029273
Zeushield	NSCLC	NA	NA	NCT03060343
PSCA/MUC1/PD-L1/CD80/86	Advanced lung or other cancers	NA	NA	NCT03198052
PSMA, FRa	Bladder cancer, urothelial carcinoma bladder	NA	NA	NCT03185468
Claudin18.2	Advanced gastric adenocarcinoma, pancreatic adenocarcinoma	NA	LV	NCT03159819

*CTX* cyclophosphamide, *FLU* fludarabine, *LV* lentiviral, *NA* not available, *NSCLC* non-small cell lung cancer, *RV* retroviral

Most CARs in the CAR-T trials in China are second-generation CAR constructs, which have one co-stimulatory signal [41]. A trial of CAR-T cells containing a third-generation CAR construct with both CD28 and CD137 co-stimulatory signals is still recruiting patients with relapsed/refractory ALL (NCT02186860). Fourth-generation CARs have incorporated additional elements in the CAR constructs, such as an inducible caspase-9 gene element that can lead to self-destruction by apoptosis of the CAR-T cells [53]. A total of 10 trials of CAR-T cells contain a fourth-generation CAR (Table 5). Among these, five trials are evaluating CARs with an inducible caspase-9 suicide switch.

**Table 5:** Clinical trials of CAR-T cells with fourth-generation CARs in China

Target antigen	Disease	Vector	NCT no.
CD19	B cell malignancies	LV	NCT03050190
CD19	B cell lymphomas	LV	NCT02247609
CD19	B cell leukemia	LV	NCT02968472
CD19/CD22	B cell leukemia, B cell lymphoma	LV	NCT03098355
CD19 and CD20/CD22/CD38/CD123	B cell malignancy	LV	NCT03125577
CD30	Lymphoma	LV	NCT02274584
PSMA, FRa	Bladder cancer, urothelial carcinoma bladder	NA	NCT03185468
EGFRvIII	Glioblastoma multiform	NA	NCT03170141
GD2	Neuroblastoma	LV	NCT02765243
GD2	Solid tumor	LV	NCT02992210

*LV* lentiviral vector, *NA* not available

The recombinant CAR cassette is typically packaged into a pseudo-lentivirus vector which can efficiently incorporate into the genome of T cells. To date, the lentiviral vector is the most commonly used vector in CAR-T cells. The other vector commonly used is the retroviral vector (Tables 2, 3, and 4).

### Antigen targets

By altering a specific antigen-targeting element, the specificity of the CAR-T cells can be easily re-directed to a specific type of malignancy. This makes the CAR-T cell therapy highly versatile. A number of antigens have been targeted in this way. More and more antigens are being engineered into CAR-T cells, leading to a large repertoire of CAR-T cells that are being explored for the therapy of both solid and hematological malignancies (Tables 3 and 4).

CD19 is the most commonly targeted antigen to date (Table 2). Out of the 121 trials, 57 trials have CD19 as a target. Currently, there are 19 clinical trials in China targeting non-CD19 antigens, including CD20, CD22, CD30, CD33, CD38, CD123, CD138, BCMA, and Lewis Y antigen for hematological malignancies (Table 3). Dual- and multi-specificity CAR-T cells have also been in clinical trials in China.

### Current trials on hematological malignancies

The most common type of diseases in CAR-T trials are B cell malignancies, including leukemia, lymphoma, and myeloma.

The CD19-targeted autologous CAR-T product, tisagenlecleucel, was recently approved by FDA for therapy of refractory/relapsed (r/r) B cell ALL. In 30 patients including children and adults who received this product, 90% of them achieved complete remission (CR) [54]. Severe cytokine-release syndrome (CRS) was reported in 27% of the patients. This product has been in clinical trials for CD19+ B cell malignancies, including CLL, ALL, and lymphoma [21–24, 54, 55]. In a Chinese study (NCT 02813837), 30 patients (5 children and 25 adults) with r/r ALL were treated with autologous CD-19 CAR-T cells [56]. In this 2017 report of preliminary results of a seven-center clinical trial, CR was 86% and severe CRS was seen in 26% of the patients [56]. Successful outcome has been reported with other CAR-T cells against CD19 antigen in r/r ALL [29, 32, 57–59].

The CD19-specific CAR-T cells, axicabtagene ciloleucel (axi-cel, KTE-C19), have been reported to be safe for treatment of aggressive lymphomas including r/r diffuse large cell lymphoma (DLBCL) [25]. In the phase II part of the ZUMA-1 trial, overall response rate (ORR) was 76% (47% CR and 29% PR) at the time of report in the cohort 1 of 51 patients [60]. This product is currently under evaluation by FDA.

CD33 and CD123 are targets on myeloid leukemias. Currently, there are three trials on CAR-T cells targeting CD33 and two trials targeting CD123 antigen in China (Table 3). In the USA, three CAR-T trials targeting CD123 were either terminated (NCT02623582) or suspended (UCART123, NCT02159495, and NCT03190278) at this time.

B cell maturation antigen (BCMA) is an antigen target on myeloma cells. Currently, three trials on BCMA-targeted CAR-T cells are being done in r/r myeloma in China (Table 3). In one of the trials of CAR-T cells targeting BCMA in China, 19 patients with r/r multiple myeloma were evaluable and 7 of the patients were followed for more than 6 months at the time of the report [61]. CRS was observed in 14 (74%) patients. The ORRs were close to 100% in the evaluable r/r myeloma patients. The outcome from the preliminary report was highly encouraging. Complete response was also reported in a case of r/r myeloma patient who received autologous CTL019 cells, even though 99.95% of the myeloma cells were negative for CD19 [38, 62]. It appears therefore that multiple myeloma is highly sensitive to immunotherapy.

There are also a few registered clinical trials that are testing two or more CARs either simultaneously or sequentially. In the trial NCT02846584, patients receive intravenously infused autologous anti-CD19 or anti-CD20 CAR-T cells to treat B cell malignancies. Another trial, NCT02737085, is to explore the sequential therapeutic effect of anti-CD19 and anti-CD20 CAR-T cells in the treatment of DLBCL.

The trial NCT02903810 was planned with a treatment scheme of infusion of equal numbers of anti-CD19 and anti-CD22 CAR-T cells in the treatment of refractory hematologic malignancies. Two trials (NCT03097770 and NCT03098355) target two antigens simultaneously with one CAR construct (Table 2). These trials are ongoing at this time.

### Current trials on solid tumors

Multiple solid tumors are being studied in CAR-T clinical trials. At the time of this report, 20 different antigens are being targeted in solid tumor trials (Table 4). GPC3, mesothelin, epidermal growth factor receptor (EGFR), and EpCAM were the most targeted antigens (Table 4). This is consistent with reports from international trials [63–68]. Liver cancer remains the most commonly studied solid tumor in China [69]. In a preliminary report of a trial of CAR-T cells against CD133+ epithelial tumors (NCT02541370), 24 patients were enrolled, including 14 patients with sorafenib-refractory hepatocellular carcinoma (HCC), 7 with pancreatic carcinomas, 2 with colorectal carcinomas, and 1 with cholangiocarcinoma [69]. The number of CAR-T cells was found to be inversely related to the CD133+ epithelial cells in peripheral blood. There was a separate report treating refractory cholangiocarcinoma with sequential infusion of two different types of CAR-T cells targeting EGFR and CD133 [70].

Two trials in China are evaluating GD2 antigen-targeted CAR-T cells in neuroblastoma (Table 4). Another two trials are evaluating CAR-T cells against EGFRvIII+ glioblastoma. There was one case report in the literature on rapidly progressing refractory glioblastoma that showed dramatic CR to IL13Rα2-targeted CAR-T cells after repeated infusion [71]. In a separate report, nine patients with refractory EGFRvIII+ glioblastoma received autologous CART-EGFRvIII cells in a pilot study [66]. Interestingly, there was no CRS observed. CAR-T cell infiltration was shown in the resected tumor specimen. This study suggested that the CAR-T cells are safe and immunologically active with tracking capability to the cancer cells in the brain.

Multiple antigens are being explored as targets in solid tumors for CAR-T cells (Table 4). Preliminary reports have been presented and published throughout the world [64, 65, 67, 72]. Outcomes from larger sample size and longer follow-up are clearly needed from these trials.

### CAR-T trials for non-malignant diseases

There is currently one clinical trial of autologous CAR-T19 cells for patients with systemic lupus erythematosus (NCT03030976, Table 2). This trial is designed to infuse 1 × 10^6^ cells/kg. More trials are expected to come for non-malignant diseases.

## Discussion

This study analyzed CAR-T trials in China. Most CAR-T trials are employing autologous T cells. CD19 is the most commonly targeted antigen. Therefore, B cell leukemia and lymphoma are the most common malignancies in CAR-T trials. Solid tumors remain a significant challenge for CAR-T therapy [45, 70, 73, 74]. Challenges include selection of target antigens, management of toxicities, and modulation of tumor microenvironment [75, 76]. Loss of CD19 expression is a known mechanism for relapse from CD19-directed CAR-T therapy [77]. The first CAR-T product, tisagenlecleucel, was recently approved. KTE-C19 for large cell lymphoma is under evaluation by FDA [25, 60]. It is unclear which product among many ongoing clinical CAR-T trials in China has independent patent that may lead to final approval for clinical application in China.

It has been well documented that CAR-T cells can cross the blood-brain barrier [23, 78, 79]. CAR-T cells may become an effective therapy for refractory CNS diseases [66, 71, 78–81]. In addition to trials of single-target CAR-T cells, simultaneous and sequential CAR-T cells are being studied for clinical applications [70]. Multi-target CAR-engineered T cells are also entering clinical trials (Tables 2, 3, and 4).

The currently approved tisagenlecleucel CAR-T therapy relies on transduction of autologous T cells from patients. It is important therefore to be able to reliably obtain and propagate adequate amount of T cells. This may become a major limitation for wide application of this new therapy. Therefore, newer CARs are being actively investigated [41, 82–84]. Universal CAR-Ts have been generated by inactivating HLA class I molecules and used successfully in patients [82, 85, 86]. Allogeneic CAR-T cells are entering clinical trials [42, 87]. T cell receptor-engineered CAR-T cells represent another frontier in CAR-T cell development [88–90]. It is foreseeable that CAR-T immunotherapy will become a major modality of cancer therapy (Table 5) [91].

## Abbreviations

ALL: Acute lymphoblastic leukemia
AML: Acute myeloid leukemia
BCMA: B cell maturation antigen
CTX: Cyclophosphamide
DLBCL: Diffuse large B cell lymphoma
FLU: Fludarabine
HL: Hodgkin’s lymphoma
LV: Lentiviral
MCL: Mantle cell lymphoma
NHL: Non-Hodgkin lymphoma
